# Termite Incidence on an *Araucaria* Plantation Forest in Teluk Bahang, Penang

**DOI:** 10.3390/insects2040469

**Published:** 2011-11-02

**Authors:** Aiman Hanis Jasmi, Abu Hassan Ahmad

**Affiliations:** School of Biological Sciences, Universiti Sains Malaysia, Minden 11800, Penang, Malaysia; E-Mail: aimanhanis87@yahoo.com

**Keywords:** termite diversity, *Araucaria* plantation forest, *Coptotermes curvignathus*, Teluk Bahang

## Abstract

A study was carried out to evaluate the incidence of termite attack on an *Araucaria cunninghamii* plantation at Teluk Bahang Forest Park (TBFP), Penang. The hilly plantation area was surveyed to determine the diversity of termite species present. Termite specimens were collected from standing *Araucaria* trees, underground monitoring (aggregation) stations, fallen logs, forest litter and mounds (nests). Seven species of termites were identified from 6 genera; *Coptotermes curvignathus*, *Schedorhinotermes medioobscurus*, *Schedorhinotermes malaccensis*, *Odontotermes sarawakensis*, *Parrhinotermes aequalis*, *Macrotermes malaccensis* and *Hospitalitermes hospitalis*. A total of 289 *Araucaria* trees were inspected for signs of termite attack. Termite infestation of trees was determined mainly by the presence of mud on the trunk, but particularly around their butts at ground line. The most dominant termite species discovered infesting the *Araucaria* trees was *Coptotermes curvignathus*; accountable for 74% of all infestations. *Schedorhinotermes medioobscurus* and *Odontotermes sarawakensis* were commonly found infesting dead trees and/or tree stumps. Approximately 21.5% of all *Araucaria* trees in the plantation forest at Teluk Bahang were infested by termites.

## 1. Introduction

Termites in the natural tropical ecosystem serve as important decomposing agents of organic matter [17]. Their feeding and tunneling activities improve porosity, aeration, stability and nutrient enrichment of the soil as well as facilitating C mineralization and N fixation [6,9,18,19]. A total of 175 species of termites from 42 genera have been recorded in Peninsular Malaysia [14].

Termites are generally known to be economically important insect pests. Su [11] has estimated that the annual worldwide cost for termite control and the associated repair of damage is USD 20 billion. In Malaysia, the cost for termite control in 2003 was approximately USD10-12 million while the cost of repairs was estimated to be much higher [8]. Termite species of the genus *Coptotermes* are considered the most economically important, responsible for almost 85% of all infestations in Malaysia [10].

Apart from being known as structural pests, termites can also infest and destroy crops and plantation trees in agro-forestry. In a number of studies, the severity and high incidence of termite attack in Malaysian conifer plantations has been highlighted [1,3,12,13]. *Coptotermes curvignathus* Holmgren, a key pest species of plantation forests, kills and damages trees of any age [14]. Buildings and structures are also vulnerable to termite infestation, especially those constructed of wood and wood products. This paper details a study conducted to ascertain the number and severity of termite infestations in conifer (*Araucaria cunninghamii*) trees within a plantation forest.

## 2. Experimental Section

The study was conducted in the Teluk Bahang Forest Park (TBFP; 5° 26′ 47″ N, 100° 13′ 06″ E) in Penang. TBFP was established in 1974 and is part of the Teluk Bahang Recreational Forest which spans about 873 hectares and is located approximately 24 km from Georgetown. TBFP (32 hectares) includes recreational areas, buildings and structures, a plantation forest of *Araucaria cunninghamii* as well as some natural rainforest. However, the *Araucaria* plantation (approximately 2 hectares) is only a small area within TBFP.

A total of 289 *Araucaria* trees were inspected for any signs of termite activity. Concurrently, observations on the condition of the trees were undertaken. These trees were categorized as healthy, in-decline, dead or stump, based on their physical condition. If present, termites were sampled from the standing *Araucaria* trees, fallen logs, forest litter and mounds (nests) within the plantation. The number of *Araucaria* trees infested by termites was recorded. Collection of specimens on *Araucaria* trees and fallen logs were made by breaking the mud-tubes and hand-picking the termite using soft forceps. Forest litter with termites were placed in plastic bags and later segregated at the laboratory. Termite mounds found within the plantation were broken to allow collection of specimens while openly foraging termites were directed picked up and placed in universal bottles containing alcohol (75%). Furthermore, termites were sampled from 15 underground monitoring (aggregation) stations (UMS) that had previously been installed at random within the plantation. Hollow plastic containers (21 cm in height, 20 cm in diameter) were utilized as the underground monitoring station with 9 *Araucaria cunninghamii* wood stakes (2.4 × 2.4 × 17.4 cm each stakes) tied together with rubber bands placed within the container. The samples of termites collected were taken to the laboratory, preserved in alcohol (75%) and later identified using the key provided by Tho [15] and Thapa [13].

## 3. Results and Discussion

Seven termite species from 6 genera were found within the plantation (Table 1). Three species (*Coptotermes curvignathus*, *Schedorhinotermes medioobscurus* and *Odontotermes sarawakensis*) were observed infesting *Araucaria* trees and were therefore designated as pest species. With the exception of *Hospitalitermes hospitalis*, all of the recorded termite species were found to be infesting the UMSs. These species are viewed as potentially harmful species as they feed on *Araucaria* wood stakes in the UMSs. *H. hospitalis* were found foraging openly on the ground in long columns within the plantation. This species of termite often consume lichens, therefore poses no threat to the trees [5,15].

In a previous study on termite diversity in TBFP, Aiman Hanis *et al.* [2] reported 16 termite species from 11 genera but the study included areas within TBFP that had been disturbed by human activity in addition to the *Araucaria* plantation described in this paper. Our study was able to identify three additional species of termite (*Schedorhinotermes malaccensis*, *Parrhinotermes aequalis* and *Macrotermes malaccensis*) in TBFP.

The total number of *A. cunninghamii* trees in the plantation were 289. It is estimated that more than 400 trees were planted at the establishment of the plantation. This estimation was based upon fallen tree trunks and the vacant spaces between the remaining trees. However, no records exist that can support this estimation.

Of all the trees originally established within the plantation (>400 estimate), only 210 remain in an apparent healthy state and appear to be free of infestation by termites while 34 were considered in-decline. The remaining were dead trees (15) and stumps (30) (Table 2). Four apparently healthy trees were recorded as being in the early stages of infestation by *C. curvignathus* indicated by the modest amount of mud tubes and soil covers on the tree trunk. All the dead trees and tree stumps inspected were actively infested by termites (Table 2).

The dominant termite species within the *A. cunninghamii* plantation was *C. curvignathus*. Forty-six infested trees were inhabited by this species representing for about 74% of the total number of infested trees. This species infested all four tree habitats (healthy, in-decline, dead and stump) (Table 2). *C. curvignathus* is known to cause damage and eventual death of trees at any age [14]. This species is a common, most important pest of agriculture and plantation forests in Malaysia. Tho and Kirton [16] reported that *C. curvignathus* was the sole pest species attacking trees in conifer plantation at which *A. cunninghamii* concluded as the most susceptible to termite attack. Cheng *et al.* [4] identified *Coptotermes spp*. as a damaging species to palm trees with *C. curvignathus* being capable of killing the trees.

Another important pest species was *S. medioobscurus* observed infesting mostly dead trees (4) and tree stumps (8). None of the living trees were infested by *Schedorhinotermes* spp. (Table 2). However, *Schedorhinotermes* spp. was observed infesting a tree that was in decline, perhaps indicating that this species may not infest living trees. This finding is in accordance with the study by Cheng *et al.* [4] on oil palm plantation suggesting *Schedorhinotermes* only infests dead tree and plant residues. Three stumps were infested by *O. sarawakensis* (Table 2).

A total of 62 *Araucaria* trees (21.5%) were confirmed to be actively infested by termites, as indicated by the presence of mud-trails on the trunks or soil covers around the butts at ground line. Extensive mud plastering around the bases of the tree trunks was the most common indicator of severe *C. curvignathus* infestation recorded by Tho and Kirton [16]. Another 21 trees declared to be “in-decline” were suspected to be infested by termites, although no evidence of activity could be found. Declining trees often indicate internal infestation of termite that caused damage to the heartwood and vascular system (cambium tissue) of these trees, a mode of infestation described as the “heartwood infestation” [7]. Five of the “in-decline” trees surveyed in the plantation were subsequently felled and all were confirmed to be infested by *C. curvignathus*.

The percentage of trees infested by termites may be greater as it is possible that some have yet to show symptoms. In some exotic plantations of *Araucaria* spp. and *Pinus* spp. in Malaysia, up to 100% of trees have been known to be infested by *Coptotermes* spp. [1]. Tho [14] reported that exotic trees growing in Malaysia were more susceptible to termite infestation than were indigenous trees. Consequently, most exotic conifer trees such as *A. cunninghamii*, *A. hunsteinii*, *Pinus caribaea* and *P. patula* experience termite damages. In view to this, termite management strategies should be implemented in existing conifer plantations to halt further damages on the trees. It is also recommended to be taken into consideration upon establishment of new plantation to avoid possible economic losses due to termite attack.

## 4. Conclusions

Seven termite species were identified within the *Araucaria cunninghamii* plantation of Teluk Bahang Forest Park (TBFP). Three species (*Coptotermes curvignathus*, *Schedorhinotermes medioobscurus* and *Odontotermes sarawakensis*) were designated as key pest species, capable of infesting *Araucaria* trees. *C. curvignathus* was the most dominant pest species accountable for about 74% of the total infestation.

## Figures and Tables

**Table 1:** Incidence of termite species on materials in *Araucaria* plantation forest.

**Termite sp.**	**Location**				
	***Araucaria* tree**	**UMS**	**Mound**	**Open foraging**	**Forest litter**
*Coptotermes curvignathus*	X	X			
*Schedorhinotermes medioobscurus*	X	X			X
*Schedorhinotermes malaccensis*		X			
*Parrhinotermes aequalis*		X			X
*Macrotermes malaccensis*		X	X		
*Hospitalitermes hospitalis*				X	
*Odontotermes sarawakensis*	X	X			

**Table 2:** Termite species found in healthy living, in-decline and dead *Araucaria* trees in Teluk Bahang Forest Park (TBFP).

**Habitat**	**Termite Frequency By Species**				
	**Replicates**	***C. curvignathus***	***S. medioobscurus***	***O. sarawakensis***	**No termite**
Healthy tree	210	4	0	0	206
In-decline tree	34	12	1	0	21
Dead	15	11	4	0	0
Stump	30	19	8	3	0
Total	289	46	13	3	227

Note: (1) A tree was considered “in-decline” when its crown was dead; (2) A tree was considered “dead” when it no longer bore green leaves or living branches.
